# Climate variability and life history impact stress, thyroid, and immune markers in California sea lions (*Zalophus californianus*) during El Niño conditions

**DOI:** 10.1093/conphys/coz010

**Published:** 2019-05-15

**Authors:** Eugene J DeRango, Katherine C Prager, Denise J Greig, Amanda W Hooper, Daniel E Crocker

**Affiliations:** 1 Department of Biology, Sonoma State University, Rohnert Park, CA, USA; 2 Department of Animal Behaviour, Bielefeld University, Bielefeld, Germany; 3 Department of Ecology and Evolutionary Biology, University of California Los Angeles, Los Angeles, CA, USA; 4 Ornithology and Mammalogy Department, California Academy of Sciences, San Francisco, CA, USA

**Keywords:** California sea lion, capture stress, El Niño, thyroid, immunoglobulin, glucocorticoid

## Abstract

Wildlife is exposed to a diverse set of extrinsic and intrinsic stressors, such as climatic variation or life history constraints, which may impact individual health and fitness. El Niño and climatic anomalies between 2013 and 2016 had major ecological impacts on the California Current ecosystem. As top marine predators, California sea lions (CSL) experienced decreased prey availability and foraging success, impacting their nutritional state. We hypothesize that chronic stress to juvenile CSL increased during the 2015–2016 El Niño and that breeding represents a period of chronic stress for adults, which impact a variety of physiological processes. We opportunistically captured and sampled juvenile CSL (female, *n =* 29; male, *n =* 38) in central California and adult male CSL (*n =* 76) in Astoria, Oregon and quantified a suite of analytes in serum as indicators of acute stress markers, metabolism and thyroid function, and adaptive immune response. We found that stress hormones and glucose were decreased in juvenile CSL during 2016 relative to 2015 and in adult male CSL after the breeding season, which may indicate chronic stress downregulating HPA (hypothalamic-pituitary-adrenal) axis sensitivity with associated metabolic impacts. Conversely, thyroid hormones for both juvenile and adult male CSL were increased, suggesting greater energetic requirements resulting from increased foraging activity during suboptimal conditions in juveniles and breeding tenure in adult males. Immunoglobulin IgG was elevated in juveniles in 2016 but reduced in adult males post-breeding. This suggests that juveniles may face immunostimulatory pressure during anomalously warm ocean environments; however, for adult males, breeding is a significant energetic cost resulting in reductions to immune function. Our results indicate that environmental conditions and life history stage may influence physiological responses in an important marine predator and a sentinel species of changing ocean ecosystems.

## Teaser

Ocean warming events, such as the recent 2015 El Niño in the California current ecosystem, and demanding life stages, such as reproduction, can cause chronic stress in sea lions. We demonstrate that stress hormones are associated with metabolism and immune markers in chronically stressed animals.

## Introduction

All organisms are exposed to a variety of stressors over the course of their lives, and some of these stressors can have an important impact on homeostasis. Extrinsic stressors arise from chemical, physical or biological factors in an animal’s external environment (Acevedo and Duffus, 2009), all of which can be influenced by climatic variation. Intrinsic stressors are often the sequela of tradeoffs inherent to specific life history strategies. Understanding how wildlife species physiologically adapt to and cope with acute and long-term disturbances to endocrine and immune systems is critical to determine the fitness consequences of natural stressors (Wingfield *et al.*, 1997, Wikelski and Cooke, 2006, Dantzer *et al.*, 2014, McCormick and Romero, 2017).

Sustained responses to chronic stressors, such as extended periods of food limitation or reproduction, are adaptive if the stressor is cyclic, predictable and there has been sufficient time for selection to shape the response to the stressor (Boonstra, 2013). For example, glucocorticoid (GC) hormones released by the hypothalamic-pituitary-adrenal (HPA) axis can be stimulatory by increasing gluconeogenesis (conversion of non-carbohydrates, such as lactate, into glucose), metabolic rate and growth (McMahon *et al.*, 1988, Sapolsky *et al.*, 2000). If unpredictable (e.g. *major* short-term environmental disturbance), continuous exposure to a stressor can result in a decrease in both basal corticosteroids and ability to cope with future or additional stressors (Rich and Romero, 2005, Romero *et al.*, 2009). It is unclear, however, how these cumulative effects manifest in wildlife species and if these sustained responses can be considered maladaptive (Boonstra, 2013).

Repeated or sustained stress can have downstream consequences on important biological functions (McEwen and Stellar, 1993).Chronically elevated GCs can alter the hypothalamus-pituitary-thyroid (HPT) axis through a variety of mechanisms (Charmandari *et al.*, 2005, Cooper and Ladenson, 2011), which over time, will suppress thyroid function and reduce energy expenditure (Danforth and Burger, 1984, Helmreich *et al.*, 2005, Chrousos, 2009). Chronic stress can also down-regulate immune response (McEwen *et al.*, 1997), facilitating an energetic trade-off between immune function and other metabolic processes (Sheldon and Verhulst, 1996, Råberg *et al.*, 1998). The adaptive immune response, represented in part by immunoglobulin (Ig) production, is highly specialized to specific pathogens and requires substantial biochemical and/or energetic reserves with high developmental costs (McDade *et al.*, 2016).

Anomalous weather and climatic events can be major drivers for shifts and disturbances to marine ecosystems (Hughes *et al.*, 2005). Short-term warming, such as the onset of an El Niño-Southern Oscillation (ENSO) event, has been extensively documented as intense local stressors to many ecologically important marine species, such as coral reefs (Claar *et al.*, 2018), seabirds (Wingfield *et al.*, 2018), marine iguanas (*Amblyrhynchus cristatus,*Romero and Wikelski, 2001) and pinnipeds (seals and sea lions, Trillmich *et al.*, 1991). Increases in sea surface temperature (SST) can reduce the distribution of nutrients via a decrease in local upwelling and consequently, a local decrease in productivity of primary producers (Trenberth, 1997) and shifts in distribution and quantity of prey species (Enfield, 2001). For pinnipeds, unpredictable perturbations from ENSO events cause alterations to diving/foraging (Crocker *et al.*, 2006, Weise and Harvey, 2008), reproductive behaviours (Soto *et al.*, 2006, Melin *et al.*, 2008), increases in stranding events due to malnutrition (Greig *et al.*, 2005, Melin *et al.*, 2009, Barcenas-de la Cruz *et al.*, 2018) and overall mortality due to reduced pup development and survival (Trillmich and Limberger, 1985, Ono *et al.*, 1987, DeLong *et al.*, 2017). Although pinnipeds are widespread and ecologically important marine predators, few studies have measured the effect that sustained exposure to intrinsic or unpredictable stressors may have on endocrine and immune responses.

Between 2013 and 2016, there were multiple disruptions to primary productivity within the marine environment of the California Current ecosystem, including two ENSO events (Leising *et al.*, 2014, Leising *et al.*, 2015, Jacox *et al.*, 2016). Starting in 2014, a large-scale warm water anomaly within the Pacific Ocean known as `the Blob’ was associated with delays to the start of coastal upwelling, resulting in an increased duration of heightened SST (Kintisch, 2015, Peterson *et al.*, 2015). An El Niño was then declared in March 2015 with peak departures from normal monthly SST occurring around December (NOAA National Centers for Environmental Information, 2015). These combined climatic events led to a collapse and distribution shift of forage fish for an abundant top predator, the California sea lion (CSL, *Zalophus californianus*; MacCall *et al.*, 2016, McClatchie *et al.*, 2016). Foraging patterns for CSL were altered and animals were likely driven farther offshore to feed (Elorriaga-Verplancken *et al.*, 2016). When forced to increase foraging effort, nursing adult female sea lions have difficulty in allocating resources towards pup development (Ono *et al.*, 1987). Additionally, body condition and glucose-dependent immune markers, IgG and IgA (Palmer *et al.*, 2015), were reduced in dependent CSL pups born during anomalous conditions of high SST in 2015 (Banuet-Martínez *et al.*, 2017), and also showed abnormalities to erythrocytes, which can further impact nutrient uptake (Flores-Morán *et al.*, 2017). This extended period presented a unique opportunity to investigate the effects of sustained stressors on CSL facing abnormally high energetic demands.

Juvenile and adult male CSL represent critical and understudied life history stages for this species. Juvenile CSL exhibit lower diving capacity compared to mature adults (Weise *et al.*, 2010, Kuhn and Costa, 2014), and likely must modulate or increase diving effort (i.e. greater duration, longer distance), or change prey types during suboptimal conditions (Costa *et al.*, 1991). During the peak reproductive season (June and July), adult males of various body size and social rank cease foraging to participate in territorial defence throughout a long breeding tenure, which can last several weeks (Riedman, 1990). Breeding therefore carries high energetic costs, and the combined timing of breeding and suboptimal climatic conditions caused by El Niño may lead to allostatic overload and reduced energy for coping (Wingfield and Ramenofsky, 2011).

**Figure 1:**
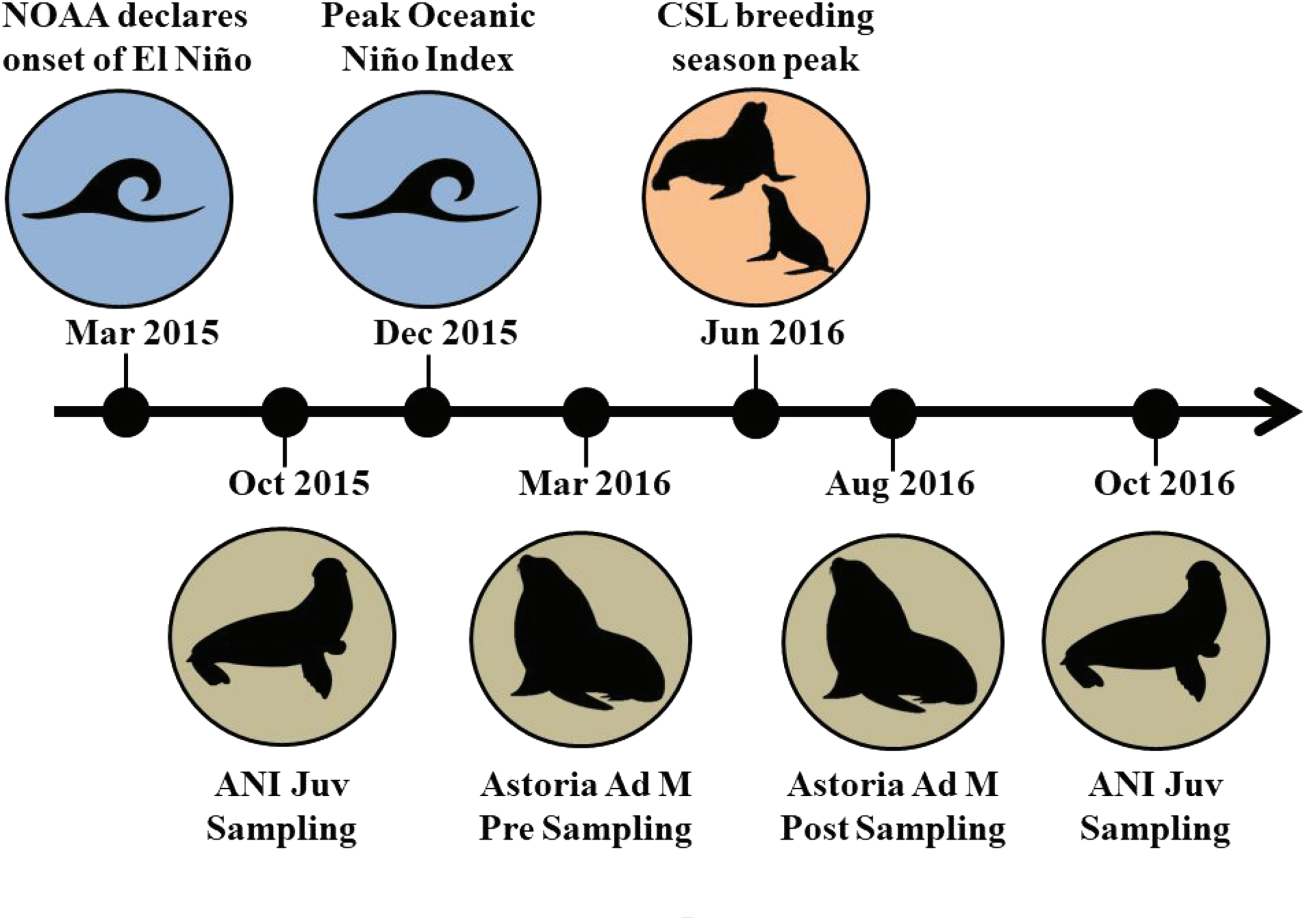
Timeline of events relevant to the CSL sampling periods during this study. During March 2015, an El Niño event was declared, with peak departures from normal monthly SST (Oceanic Niño Index) occurring around December 2015. Juvenile (Juv) CSL were sampled on Año Nuevo Island (ANI) during October 2015 and October 2016. Adult male (Ad M) CSL were sampled in Astoria, Oregon in March 2016 and late August 2016, both pre- and post-breeding season. NOAA data taken from State of the Climate, Global Climate Report (https://www.ncdc.noaa.gov/sotc/global/201513).

Our objective was to examine how markers of stress, metabolism and immune defence may be impacted by inter-annual differences in environmental conditions in juvenile CSL and life history stage (i.e. pre- and post-breeding) in adult male CSL. We use the 2015 El Niño event as a proxy for a variety of documented changes in primary productivity and prey availability, which likely caused chronic stress in CSL. In this study, we assessed a suite of analytes during capture (an acute stressor) in free-ranging juvenile CSL during the fall months of 2015 and 2016 in central California and adult males during the 2016 spring (pre-breeding) and late summer (post-breeding) seasons in Astoria, Oregon (Fig. 1). Specifically, we measured serum concentrations of stress hormones (total cortisol, corticosterone and aldosterone); thyroid hormones (total T4, T3 and rT3) to evaluate thyroid function; glucose and lactate as indicators of carbohydrate metabolism; and two important immune markers, IgM (novel or recent infections) and IgG (strong secondary immune response after repeat exposures to pathogens). We hypothesize that the cumulative effects of anomalous conditions surrounding the 2015 El Niño, one of the strongest on record (Jacox *et al.*, 2016), carried into 2016. Therefore, we predicted that sustained stress from energetically costly events, such as increased foraging effort and reproduction, would cause downregulation of the HPA axis in both age groups, with subsequent suppression of corticosteroids, glucose levels, thyroid hormones and immune markers across years and seasons.

## Materials and methods

### Study subject and capture procedure

We sampled individual juvenile CSL in October 2015 (female, *n* = 16; male, *n* = 17) and October 2016 (female, *n* = 13; male, *n* = 21) at Año Nuevo Island (ANI) in San Mateo County, California (37.1086°N, −122.3362°W) and adult male CSL in March (pre-breeding, *n* = 34) and late August–September 2016 (post-breeding, *n* = 42) in Astoria, Oregon (46.19447°N, −123.80292°W) as part of a larger health assessment study. Juvenile CSL at ANI were individually captured with modified hoop nets by several personnel who approached multiple animals along the beach. Once captured, CSL were placed into separate carriers (Petmate Vari Kennel, dimensions 48" L X 32" W X 35" H) ~25 m from the sampling area. Single CSL were successively removed, recaptured, physically restrained, anesthetized with isoflurane gas and sampled. Adult CSL at Astoria voluntarily hauled out on and confined in a floating cage trap, i.e. a modified large, metal capture cage with sliding guillotine doors set open. Individual CSL were ultimately led into a squeeze cage into which animals were moved for sedation with a combination of midazolam (0.1 mg/kg) and butorphanol (0.1 mg/kg) and anaesthesia using isoflurane gas. The methodology for adult captures is described in greater detail by Wright *et al.* (2010) and Melin *et al.* (2013).

Once anaesthesia had begun, blood was collected as soon as safely possible from the caudal gluteal vein via Vacutainer needles into 10 ml serum Vacutainer tubes. Times of capture, anaesthesia start and blood collection were recorded, as were the sex and standard length (SL). SL was measured as a straight length from the tip of the nose to the tip of the tail. Ages of juveniles were estimated to be between 1.5 and 4.5 years old based on methods with known age individuals using a combination of body and tooth size, and lack of secondary sexual characteristics (Greig *et al.*, 2005, Laake *et al.*, 2016). Adult males were identified and aged based on size, pelage colour and presence of a fully developed sagittal crest. Due to logistical difficulties, we were unable to measure body mass in all animals in this study and instead used SL as a proxy for body size. Before release, all CSL were given a numerical front flipper tag (Allflex USA, Inc.) to identify individuals; and there was no repeated sampling of individuals.

### Sample analysis

Once collected, blood samples were allowed to clot and centrifuged that day at 1534 *g* (LW Scientific Portafuge E7, USA) for 15 minutes to separate serum. Samples were frozen and stored at −80°C until analysis. We quantified total concentrations of all analytes in duplicate using commercially available assay platforms. Mean concentrations for each analyte separated by age class and sampling period are found in Supplementary Tables 1 and 2. Cortisol, total T3 (TT3), total T4 (TT4) (MP Biomedicals, Orangeburg, NY, USA), rT3 (ALPCO, Salem, NH, USA) and aldosterone (IBL International, Hamburg, Germany) were measured using hormone specific I^125^ radioimmunoassay (RIA) kits. Corticosterone was measured using enzyme-linked immunosorbent assay (Cayman Chemical Company, Ann Arbor, Michigan, USA). IgG and IgM were measured using antibody-sensitized microsphere microagglutination assays (Thermo Fisher Scientific, Waltham, MA, USA). Metabolites glucose and lactate were measured using a YSI 2300 analyzer to yield quantitative (i.e. mM) rather than relative values. GCs and aldosterone were measured in all serum samples (*n =* 143), and thyroid hormones, Igs and metabolic markers were measured in a subset of animals depending on available sample volume (TT3, *n =* 137; rT3, *n =* 108; and TT4, *n =* 115; IgG and IgM, *n* = 102; lactate/glucose, *n =* 103).

**Figure 2:**
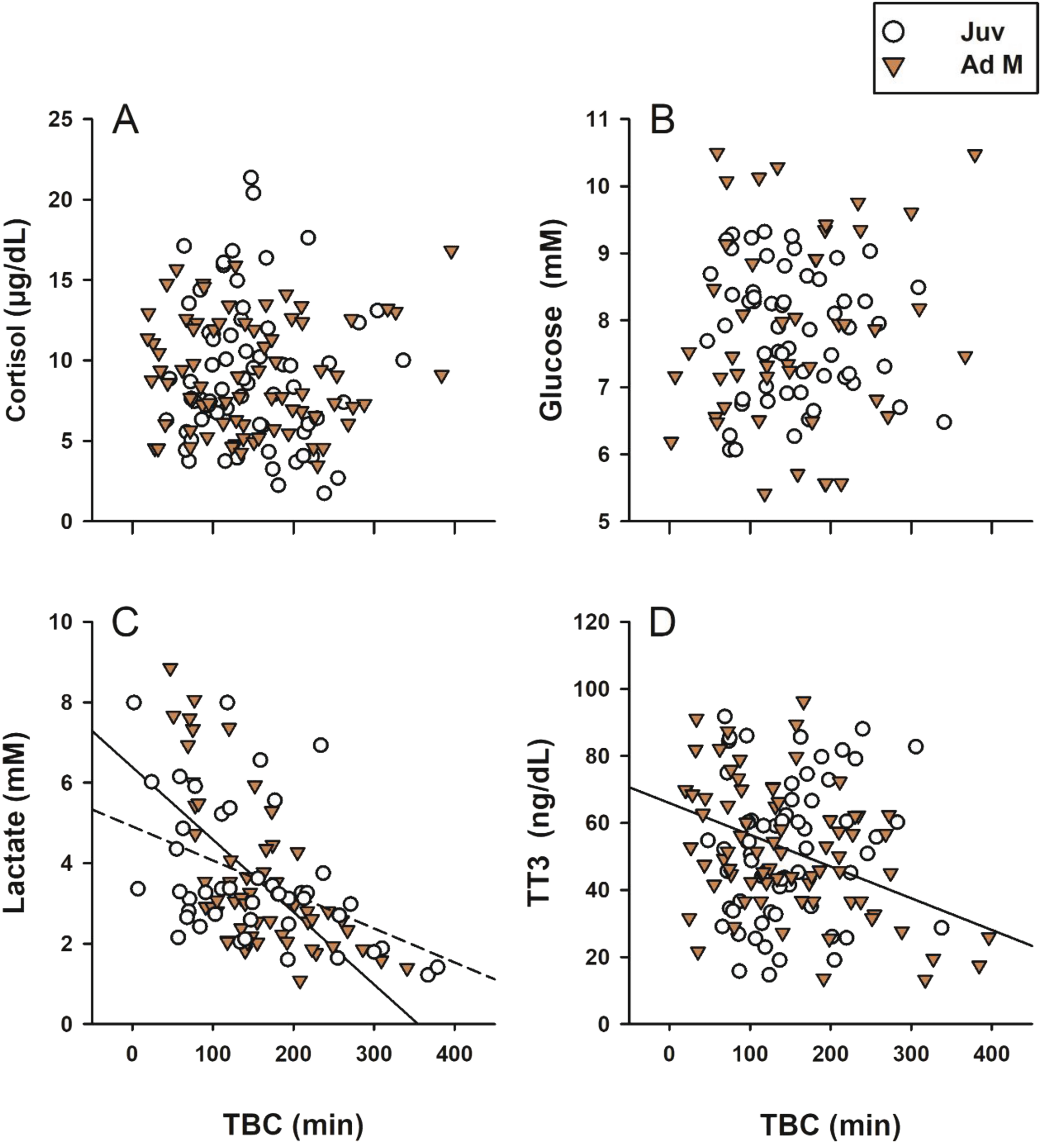
Relationships between length of time between capture and blood collection (TBC) and stress and metabolism markers in juvenile (Juv) and adult male (Ad M) sea lions. Separate lines of fit (Juv, dotted; Ad M, solid) are derived from GLM leverage plots when age class was included as a fixed effect. TBC exhibited no significant linear relationships with cortisol (a) or glucose (b) concentrations (*P >* 0.05). Lactate (c) was initially high but significantly decreased with TBC in both age classes (Juv, R^2^ = 0.40, *P <* 0.0001; Ad M, R^2^ = 0.20, *P =* 0.002), and TT3 (d) decreased with time in adult males only (*R^2^* = 0.19, *P* < 0.0001).

Initial values for serum cortisol, aldosterone, TT3 and rT3 were on the standard curve provided with manufacturer’s standards. Other analytes were diluted according to the following methods to be brought onto the standard curve: corticosterone samples diluted 1:10 with EIA buffer, TT4 samples diluted 1:3 with zero calibrator and both IgM and IgG diluted 1:240 with manufacturer provided dilution buffer. All assay platforms for GCs, aldosterone, thyroid hormones and Igs were validated for use in CSL. Serially diluted samples for each analyte yielded curves that were parallel to the standard curves (Supplementary Figures 1 and 2). We assessed accuracy and calculated % mean recovery of three added standards for each assay platform (cortisol = 96.5%, corticosterone = 96.3%, aldosterone = 103.1%, TT3 = 97.1%, rT3 = 98.9%, TT4 = 101.2%, IgG = 97.2%, IgM = 102.1%). Mean intra-assay coefficient of variation (CV) was calculated for each analyte (cortisol = 2.43%, corticosterone = 2.67%, aldosterone = 2.28%, TT3 = 1.88%, rT3 = 3.13%, TT4 = 1.6%, IgG = 5.61%, IgM = 3.43%, lactate = 1.33%, glucose = 1.56%). All assays were performed in single runs except rT3. Mean inter-assay CV for rT3 was 3.38%.

### Statistical analysis

To account for the large variability in handling duration, we first evaluated the effect of time between initial capture and blood collection (hereafter TBC) on each analyte using a general linear model (GLM) with TBC and age class as independent variables. TBC was included as a covariate in subsequent models when significant relationships were found for the respective analyte. Model *R^2^* values for TBC were derived by plotting the GLM effect leverage pairs for each analyte accounting for the effect of age (*y* = partial residuals, *x* = regressor shrinkage). We then created scatterplot matrices and investigated significant associations between measured analytes using multivariate Pearson correlations for each age class (Supplementary Figure 3 and 4). Finally, because of differences in capture techniques, geographic locations and time periods for adults and juveniles, we did not attempt to make specific comparisons for temporal effects between the age classes. We instead used two separate GLMs to independently examine how analytes changed with year, sex and body size in juvenile CSL, and between pre- and post-breeding seasons and with body size in adult male CSL. For juveniles, sex, year and SL were included as fixed effects. Season and SL were included for adult males. When significant differences between groups were detected, *post hoc* comparisons were performed using Student’s *t*-tests. For all tests, model residuals were visually assessed for normality and homoscedascity, and results were considered significant when *P <* 0.05. Data analyses were performed with JMP Pro 13 (SAS Institute, Cary, NC, USA).

**Figure 3:**
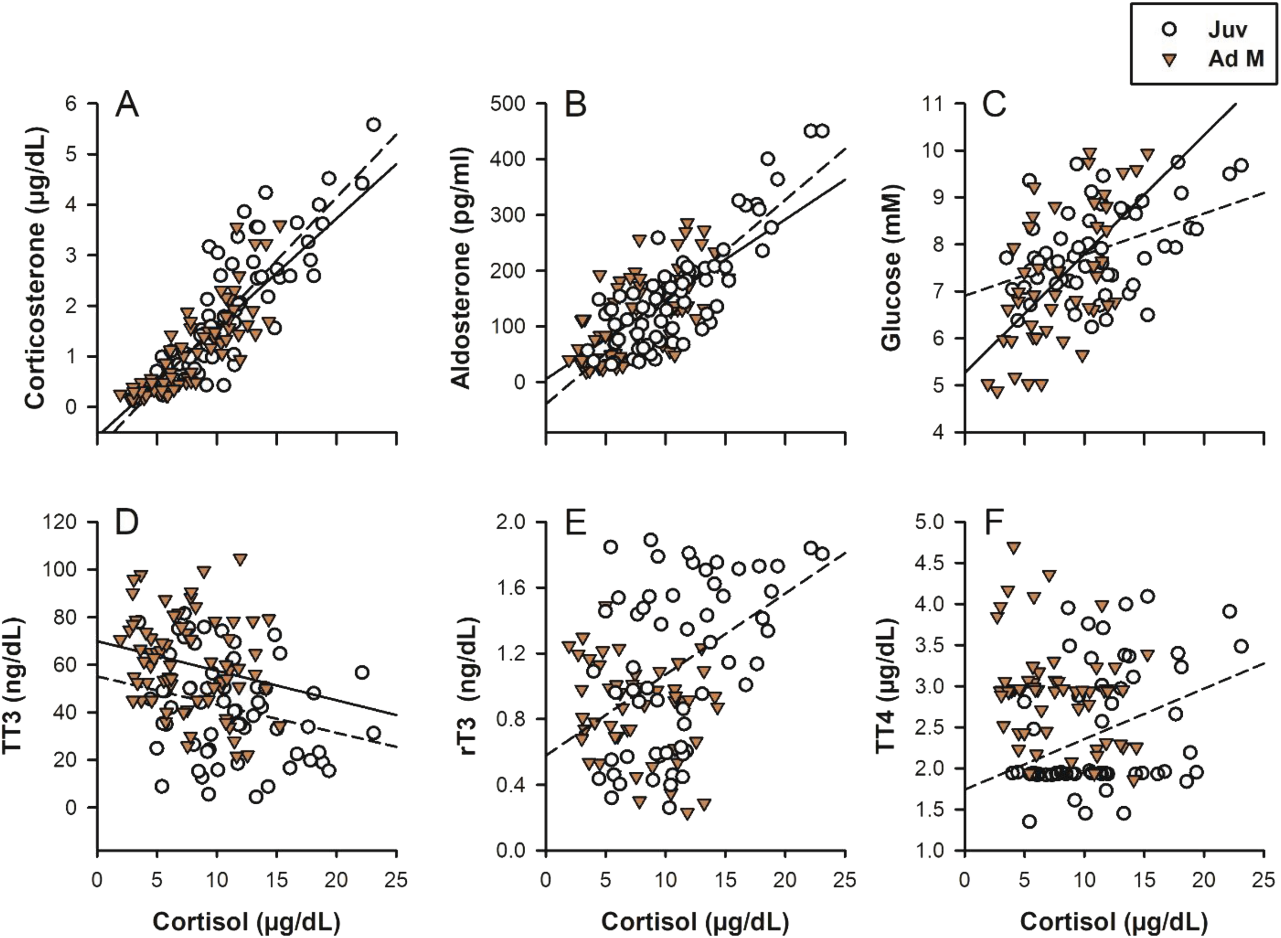
Relationships between markers for stress and metabolism in juvenile (Juv) and adult male (Ad M) sea lions. Separate lines of fit (Juv, dotted; Ad M, solid) are derived from Pearson’s correlations using multivariate comparisons for each age class. Cortisol was significantly positively associated with corticosterone (a, Juv, r = 0.86, *P* < 0.0001; Ad M, r = 0.85, *P* < 0.0001), aldosterone (b, Juv, r = 0.83, *P* < 0.0001; Ad M, r = 0.67, *P* < 0.0001) and glucose (c, Juv, r = 0.39, *P =* 0.0009; Ad M, r = 0.67, *P =* 0.0009), and negatively associated with TT3 (D, Juv, r = −0.14, *P =* 0.0133; Ad M, r = −0.27, *P =* 0.0044). rT3 (E, r = 0.45, *P =* 0.0007) and TT4 (F, r = 0.51, *P =* 0.009) were positively associated with cortisol in juvenile CSL only.

## Results

### Response to capture

TBC was highly variable for juveniles on ANI (mean = 116 minutes ±65 SD) and adult males in Astoria (mean=172 minutes ±88 SD). However, times between anaesthesia start and blood collection were short by comparison for both locations (ANI, mean = 7 minutes ±5 SD; Astoria, mean = 13 minutes ±4 SD). Controlling for age class, TBC was not associated with cortisol (Fig. 2a, juveniles, *P =* 0.57; adults, *P =* 0.83), corticosterone (juveniles, *P =* 0.86; adults, *P =* 0.47), aldosterone (juveniles, *P =* 0.29; adults, *P =* 0.41), glucose (Figure 2b, juveniles, *P =* 0.36; adults, *P =* 0.29), IgM (juveniles, *P =* 0.86; adults, *P =* 0.69) or IgG (juveniles, *P =* 0.99; adults, *P =* 0.52). We therefore excluded TBC in subsequent GLM analyses for these analytes due to lack of significance and ability to explain model variance. Controlling for age class, we did, however, find a significant negative association of TBC and lactate (Fig. 2c, juveniles, *R^2^* = 0.40, *F*_1,55_ = 36.8, *P <* 0.0001; adults, *R^2^* = 0.20, *F*_1,44_ = 11.1, *P =* 0.002). Lactate was initially high in animals sampled quickly after capture but significantly lower in those sampled later. TBC did affect thyroid hormones differently between age classes. TBC did not affect TT4 (juveniles, *P =* 0.32; adults, *P =* 0.75) or rT3 (juveniles, *P =* 0.53; adults, *P =* 0.64) for either age class; however, TBC was negatively associated with TT3 in adult males (Fig. 2d, *R^2^* = 0.19, *F*_1, 73_ = 16.8, *P <* 0.0001) but not in juveniles (*P =* 0.35).

**Table 1:** Results from GLM examining effects of year, sex and standard length (SL) on analytes in juvenile CSLs

	*Den df*	Year	*F*	*P*	Sex	*F*	*P*	SL	*F*	*P*
Cortisol	63	**2016 < 2015**	23.1	**<0.0001**	**F > M**	4.89	**0.031**		0.87	0.35
Corticosterone	*63*	**2016 < 2015**	19.7	**<0.0001**		0.37	0.55		0.13	0.72
Aldosterone	*63*	**2016 < 2015**	15.3	**<0.0001**		2.14	0.15		0.001	0.98
Total T4	52	**2016 < 2015**	4.23	**0.045**	**F > M**	5.48	**0.023**		1.36	0.25
Total T3	*58*	**2016 > 2015**	22.8	**<0.0001**	**F > M**	22.7	**<0.0001**		0.23	0.63
Reverse T3	*53*	**2016 < 2015**	23.3	**<0.0001**		0.03	0.86		2.97	0.09
IgG	48	**2016 > 2015**	7.06	**0.0107**		0.50	0.48		0.06	0.80
IgM	*48*		0.13	0.72	**F > M**	3.12	**0.0437**	**Neg**	7.6	**0.0081**
Glucose	*53*	**2016 < 2015**	6.54	**0.0135**		3.67	0.06		0.04	0.83
Lactate*	*52*		1.1	0.29		0.68	0.41		2.14	0.15

Significant results are in bold; inequalities denote directionality of differences via *post-hoc* Student’s *t*-tests. Denominator degrees of freedom (den df) were calculated via GLM output. *We controlled for time of blood collection (TBC) for lactate.

**Figure 4:**
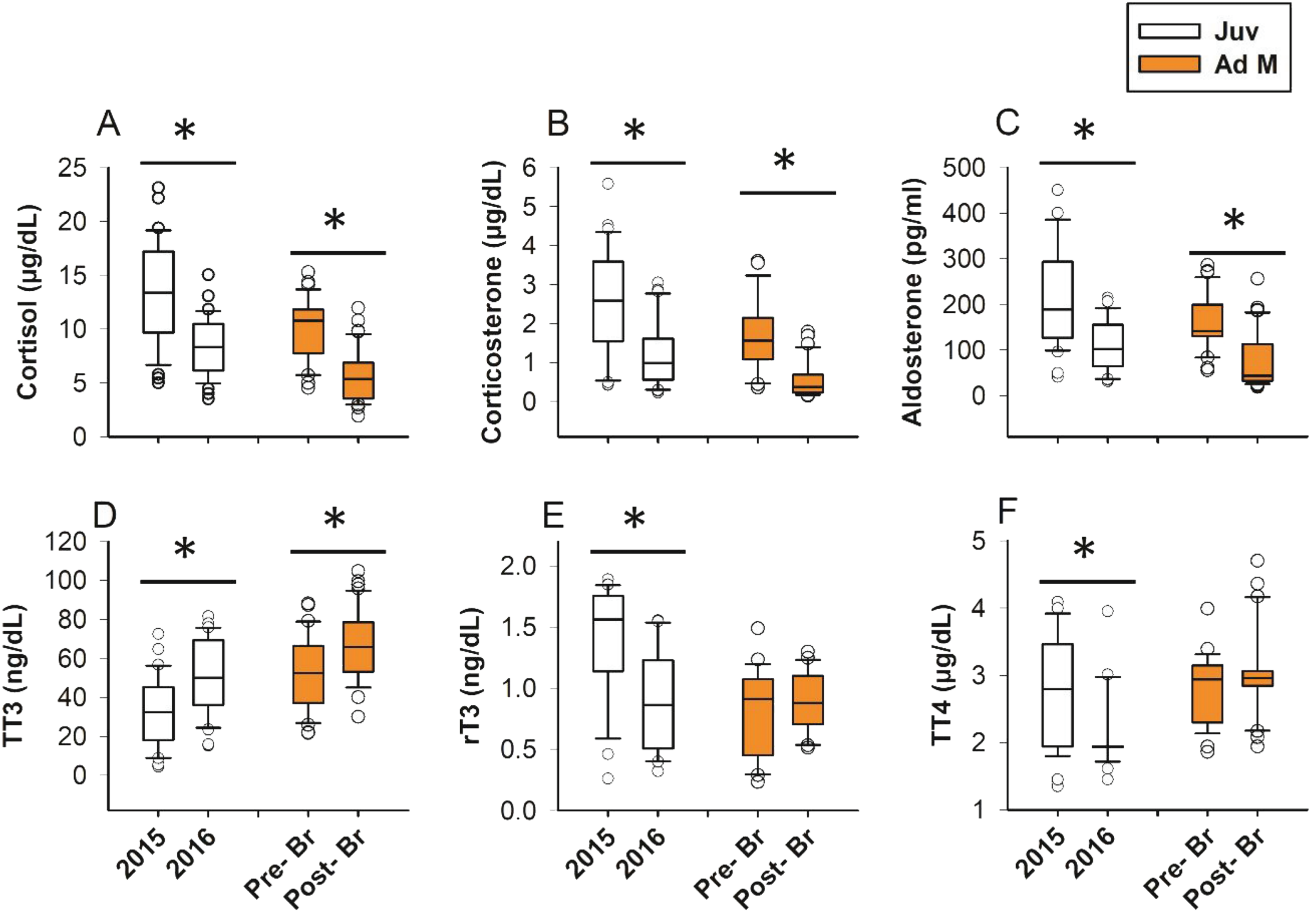
Box plots depicting stress and thyroid hormone concentrations across the years 2015 and 2016 in juveniles (Juv) and the 2016 summer breeding season in adult males (Ad M). Pre- Br: pre-breeding season; Post- Br: post-breeding season. Central lines in boxes are medians, and whiskers are 95% confidence intervals with outliers. *grouped by a horizontal line denotes significant differences between years or seasons separately for each age class according to *post-hoc* Student’s *t*-tests (*P <* 0.05).

### Associations between hormones, immune markers and metabolites

We highlight significant relationships of varying directions between cortisol and other analytes (Fig. 3). Several correlations were shared for both age classes. Serum cortisol-corticosterone (juveniles, r *=* 0.86, *P <* 0.0001; adults, r *=* 0.85, *P <* 0.0001) and cortisol-aldosterone (juveniles, r *=* 0.83, *P <* 0.0001; adults, r *=* 0.67, *P <* 0.0001) were strongly and positively correlated with each other in both age classes (Fig. 3a, b). Cortisol was positively correlated with glucose (Fig. 3c, juveniles, r *=* 0.39, *P =* 0.0009; adults, r *=* 0.67, *P =* 0.0009) and negatively correlated with TT3 in both age classes (Fig. 3d, juveniles, r *= −*0.14, *P =* 0.048; adults, r *= −*0.27, *P =* 0.044). Controlling for time, cortisol was negatively associated with lactate in adult males only (r *= −*0.48, *P =* 0.017; juveniles, *P >* 0.05). Cortisol was positively correlated with rT3 (r *=* 0.45, *P =* 0.0007) and TT4 (r *=* 0.51, *P =* 0.0091) in juveniles only (Fig. 3e). We found no correlations between any corticosteroids and IgM or IgG for either age class (Supplementary Figure 3, *P >* 0.05). IgG and IgM were negatively correlated in adult males (r *= −*0.20, *P =* 0.011) and approaching significance for juveniles (r *= −*0.24, *P =* 0.08). Glucose was positively correlated with IgG for adult males (r *=* 0.40, *P <* 0.0001).

### Annual, sex and body size differences in juveniles

**Figure 5:**
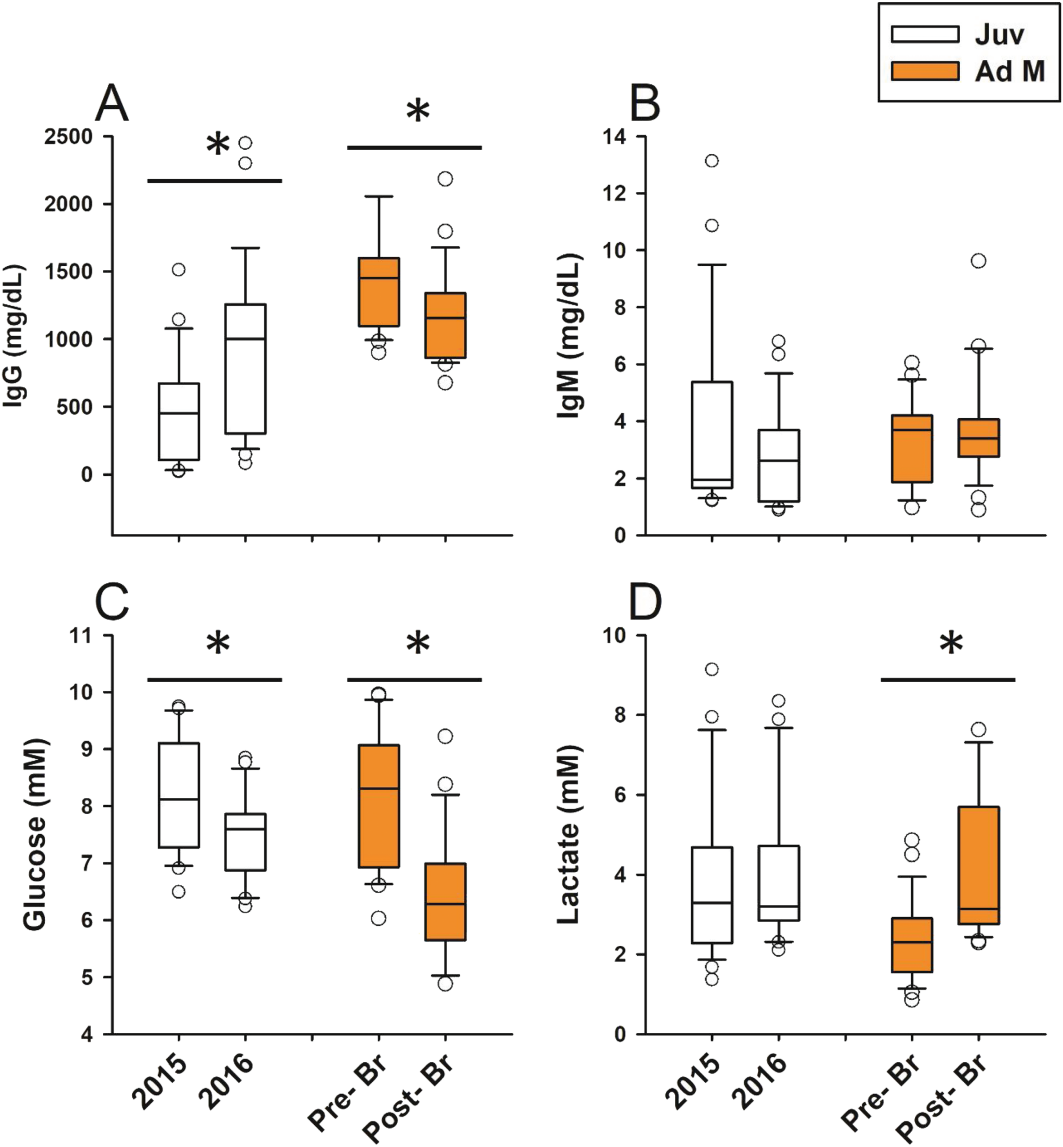
Box plots depicting Ig and glucose/lactate concentrations across the years 2015 and 2016 in juveniles (Juv) and the 2016 summer breeding season in adult males (Ad M). Pre- Br: pre-breeding season; Post- Br: post-breeding season. Central lines in boxes are medians, and whiskers are 95% confidence intervals with outliers. *grouped by a horizontal line denotes significant differences between years for Juv or seasons for Ad M according to *post-hoc* Student’s *t*-tests (*P <* 0.05).

Several strong effects of year and sex were evident for most measured analytes in juvenile CSL (Table 1). Year greatly affected all corticosteroids measured, with ~40% reductions to cortisol and ~50% reductions in corticosterone and aldosterone during 2016 compared to 2015 (Fig. 4a, b, c). Similarly, thyroid hormones rT3 and TT4 (Fig. 4e, f) and glucose (Fig. 5c) were significantly lower in 2016 compared to 2015. Conversely, we observed ~40% increases to TT3 (Fig. 4d) and IgG (Fig. 5a) in 2016 compared to 2015. IgM did not change between years (Fig. 5b). Sex only affected cortisol, TT3, TT4 and IgM (Table 1), each being significantly higher in juvenile females than males (Student’s *t*-test, *P <* 0.05). SL in juveniles only affected IgM, with smaller juveniles exhibiting elevated IgM (Table 1). As previously mentioned, lactate was greatly influenced by TBC, and year, sex and SL did not significantly affect lactate when TBC was included in the model (*P >* 0.05).

### Seasonal and body size differences in adult males

We also observed strong seasonal effects on several measured analytes in adult male CSL (Table 2). Season greatly affected all corticosteroids measured, with 45%, 67% and 55% reductions in cortisol, corticosterone and aldosterone, respectively, post- versus pre-breeding season (Fig. 4a, b, C). IgG (Fig. 5a) and glucose (Fig. 5c) were also reduced post- versus pre-breeding seasons. Conversely, when we controlled for TBC, TT3 was significantly higher post-breeding than pre-breeding (Fig. 4d, Student’s t-test, *P <* 0.05). Season had no effect on IgM, rT3 or TT4 (*P >* 0.05). SL only significantly affected cortisol and corticosterone, with smaller adult males exhibiting increased concentrations of GCs during capture. When we controlled for TBC, season (*F*_2, 43_ = 9.52, *P =* 0.0036) but not SL (*P =* 0.32) affected lactate, with lactate being higher post-breeding compared to the pre-breeding season (Student’s *t*-test, *P <* 0.05).

**Table 2:** Results from GLM examining effects of season and standard length (SL) on analytes in adult male CSLs

	*Den df*	Season	*F*	*P*	SL	*F*	*P*
Cortisol	73	**Pre > Post**	68.6	**<0.0001**	**Neg**	8.33	**0.0051**
Corticosterone	73	**Pre > Post**	57.0	**<0.0001**	**Neg**	7.67	**0.0071**
Aldosterone	73	**Pre > Post**	37.2	**<0.0001**	-	0.27	0.61
Total T4	55	-	2.77	0.10	-	0.20	0.66
Total T3*	72	**Pre < Post**	4.6	**0.0362**	-	0.01	0.91
Reverse T3	48	-	0.98	0.32	-	1.85	0.18
IgG	47	**Pre > Post**	7.06	**0.0107**	-	0.0004	0.98
IgM	47	-	0.88	0.35	-	0.41	0.52
Glucose	47	**Pre > Post**	6.7	**0.013**	-	0.15	0.71
Lactate*	42	**Pre < Post**	9.5	**0.0036**	-	1.03	0.32

Significant results are in bold; inequalities denote directionality of differences via *post-hoc* Student’s *t*-tests. Den df were calculated via GLM output. *We controlled for TBC for total T3 and lactate.

## Discussion

We found strong inter-annual and seasonal variation in several physiological markers measured after capture and prolonged restraint (a proxy for an acute stress event) in free-ranging CSL. These changes manifested as large reductions to stress hormones between sampling periods and varying directions of markers for metabolism and immune function depending on age class. The ambiguity of multiple causes of anomalous oceanographic conditions during the current study period makes the specific timeline and duration of these effects difficult to measure. Both study years (2015 and 2016) were likely stressful to CSL; therefore, we lack information on baseline stress responses but are able to compare 2 years that reflect a continuation of chronically stressful oceanographic conditions characterized by impacts to sea lion nutritional resources (McClatchie *et al.*, 2016). Nevertheless, we observed clear inter-annual variability in several aspects of the physiology of juvenile sea lions between the start of a recently declared El Niño event in 2015 and again 1 year later after exposure to increasingly high SST. We similarly observed marked changes to several markers measured in adult male CSL preceding and immediately following the reproductive season of 2016 (approximately a 6-month period). We should note that although adult sea lions in this study likely engaged in breeding behaviour; they also in general had the opportunity to forage. Adult male CSL demonstrate behavioural shifts to foraging and diving effort during El Niño conditions (Weise *et al.*, 2006) as well. Therefore, we cannot rule out the potential for temporal effects of climate variability between breeding seasons exacerbating natural life history variability for this age group.

### Handling effects on hormones and metabolism

In our study, sea lions experienced a capture event and were subsequently held in enclosures for highly variable lengths of time before being quickly recaptured and sampled. We assume here that capture acts as an acute stressor such that we expect to see physiological responses typical of short-term challenges (DeRango *et al.*, 2018, Chinn *et al.*, 2018) while recognizing that we could not standardize the timing or magnitude of the stressor. Stress hormones released during acute stressors can quickly impact metabolic pathways (Charmandari *et al.*, 2005, Champagne *et al.*, 2012, Champagne *et al.*, 2018). Therefore, within the relatively brief time frame of capture in our study, we expected increases in serum concentrations of corticosteroids, glucose and lactate post-capture, but not in thyroid hormones, which have not shown short-term changes in response to acute stress in previous studies on pinnipeds (McCormley *et al.*, 2018). Serum corticosteroids measured throughout capture via serial sampling in Guadalupe fur seals begin to decline from peak values at around 60 minutes post-capture (DeRango *et al.*, 2018). Although we found no linear relationships between holding time and corticosteroids in the current study, capture likely influenced GCs and aldosterone measured at the time of sampling. Both GCs were highly associated with aldosterone, which provides further evidence of the regulation of aldosterone secretion in association with the HPA axis in otariids as reported in other pinnipeds (Ensminger *et al.*, 2014, Champagne *et al.*, 2015, Jelincic *et al.*, 2017, DeRango *et al.*, 2018, McCormley *et al.*, 2018). We also found that TT3 was significantly lower in adult male CSL, which were held for long holding periods before sampling. Adult male elephant seals during the breeding and moulting season alter thyroid hormones during sustained exposure to GCs within 2 hours via adrenocorticotropic hormone (ACTH) challenges (Ensminger *et al.*, 2014). Therefore, the 4–6 hour holding period for some adult male CSL in our study may alter the HPT axis via sustained stress.

Most knowledge of gluconeogenesis in pinnipeds comes from studies of fasting physiology in the northern elephant seal (*Mirounga angustirostris,*Champagne *et al.*, 2013). In elephant seals there was no relationship between cortisol concentrations after restraint and rates of glucose production (Champagne *et al.*, 2012); however, it remains unknown how capture stress impacts carbohydrate metabolism in other pinniped species or otariids. In our study, glucose remained relatively high and stable (mean = 7.8 ± 1.2 mM), with no association between time to blood collection after capture. Endogenous glucose production increases with prolonged activity (Mourtzakis *et al.*, 2006); however, juvenile sea lions in this study were quickly captured after a brief but intense chase period and then remained in carriers, and adult were confined in a cage with conspecifics until sampling. Interestingly, lactate exhibited a strong relationship to handling time, where lactate was very high in animals that were sampled quickly after capture and then significantly lower in animals that were held longer. Therefore, it is likely that high lactate concentrations after the intense chase or agitation period are converted to glucose via gluconeogenesis during the rest period to keep glucose levels stable.

### Climatic and life history contexts

Lower corticosteroids measured in response to capture often reflect reduced HPA sensitivity after sustained stressors (Rich and Romero, 2005). Reductions in corticosteroids and glucose during 2016 in juvenile CSL may be consistent with suppression of the HPA axis via negative feedback secondary to chronic stress. A comprehensive analysis of Galápagos sea birds found that, generally, species showed the strongest response to induced acute stressors when their prey sources were more affected by El Niño (Wingfield *et al.*, 2018), suggesting that nutrient limitation likely plays a role in reducing HPA sensitivity. Similarly, reductions in GC release during capture in sea otters were strongest in reproductive, lactating females and those living in prey-limited populations (*Enhydra lutris*, Chinn *et al.*, 2018). We propose that the prolonged energetic stress of increased foraging effort or reduced food intake in juveniles and breeding tenure in adult male CSL may have lessened the animals’ ability to mount a normal stress response to handling (Cockrem and Silverin, 2002), evident by decreased corticosteroids measured in both age classes. Furthermore, both corticosteroids and glucose measured during capture were lower in juvenile females and smaller adult CSL. To our knowledge, only Pedernera-Romano *et al.* (2010) have measured serum GCs in free-ranging CSL, and they reported similar sex and body size effects on cortisol release in pups. As a result of decreased GCs, we expected numerous effects to downstream metabolic and immune processes.

As previously mentioned, chronically high concentrations of GCs will suppress thyroid (Ferguson and Peterson, 1992) and modulate energy reserves during stressful events. Although the effects of chronic stress on thyroid hormones are largely unexplored within CSL, we expected suppression of thyroid after chronic elevation of GCs, such as across years during anomalous conditions or after the breeding season. Low magnitude cortisol responses to handling consistent with chronic stress have been associated with a reduction in thyroid hormones in fur seals (DeRango *et al.*, 2018). Similarly, suppression of thyroid TT3 was observed after several days of repeated ACTH stimulation in fasting elephant seals (McCormley *et al.*, 2018). Because we are suggesting that reduced GCs after capture may reflect sustained stress exposure and reduced HPA sensitivity, we predicted a positive relationship between GCs and thyroid hormones. Contrary to our prediction, cortisol after capture in this study was negatively associated with TT3, and TT3 production increased across what we considered energetically costly time periods in both age classes. The observed elevation of TT3 and reductions to the reservoir hormone TT4 and inactive rT3 between years may reflect the high rates of energy expenditure found in these animals. Within pinnipeds, some life history stages may require elevation of active thyroid function during stress to support energy intensive behaviours, such as the development of foraging (Somo *et al.*, 2015) or breeding (Crocker *et al.*, 2012a) while fasting. Physiological limitations due to smaller body size may restrict juvenile CSL in our study area to small home range sizes (McHuron *et al.*, 2018a), and the spatial abundance of forage fish during the study period was limited (McClatchie *et al.*, 2016). Foraging otariids continuously maintain high field metabolic rates (Costa, 1991), and confinement to areas of reduced prey availability leads to increases in energy requirements and metabolic rates (McHuron *et al.*, 2017) and physiological challenges to juvenile CSL. Although we were not able to determine exact age or breeding success within individual adult males in this study, we also found increases in thyroid hormones measured directly after the conclusion of the breeding season. Investigations into seasonal effects on thyroid hormones in Steller sea lions (*Eumatopias jubatus*) similarly found that animals had increased concentrations during the reproductive months of July to late summer (Myers *et al.*, 2010). Adult male elephant seals when breeding maintain high rates of energy expenditure despite fasting during a long reproductive tenure (Crocker *et al.*, 2012b). The ability to raise T3 over the breeding period was associated with high rates of energy expenditure and breeding success (Crocker *et al.*, 2012a). Our results suggest that adult male CSL may upregulate T3 in a similar fashion as they fast over the duration of the breeding tenure and engage in energetically costly behaviours. It is unclear whether maintaining increased thyroid levels long term while constrained by anomalous conditions or additional unpredictable stressors has the potential to be deleterious or active an ‘emergency life history state’ (Romero and Wingfield, 2015) in these animals.

The relationship between immune function and stress related to environmental or life history variables can be complex and likely mediated by a variety of factors. IgG production is mediated by glucose, through T-cell activated class switching (Palmer *et al.*, 2015); therefore, adaptive immune responses are known to be compromised by nutrient limitation (Martin *et al.*, 2007). Reproduction is one of the most energetically expensive processes in pinnipeds (Trillmich, 1990), and reduced IgG and compromises to immune function have been found in lactating elephant seals (Peck *et al.*, 2016) and harbour seals (*Phoca vitulina,*Ross *et al.*, 1993). Although it is difficult to distinguish the breeding costs of adult male and female pinnipeds, our results suggest similar trends, which reflect physiological stress-induced immunosuppression characteristic of reproduction and energetic limitations in males. Although we found no correlations between cortisol and immune markers, adult male CSL exhibited reduced cortisol, glucose and IgG after energetically costly events during the breeding season. Adult males during colonial breeding engage in strenuous fighting with conspecifics and often inflict wounds, potentially associated with high pathogen exposure. These obligate interactions and repeat exposures to the same pathogens may create an energetic trade-off moving resources away from the adaptive immune response and towards reproduction during the breeding season.

Banuet-Martínez *et al.* (2017) hypothesized that El Niño conditions would drive nutrient limitation and trade-offs to immune function in CSL. Contrarily, IgG measured in juvenile CSL was elevated during 2016, even with observed reductions to glucose during this year. A previous study demonstrated that closely related Galápagos sea lion (*Zalophus wollebaeki*) juveniles living near human-impacted colonies had elevated IgG responses, likely due to increased pathogen exposure from a myriad of environmental sources (Brock *et al.*, 2013a). Our pattern similarly may reflect greater immunostimulatory pressure with individuals mounting a large immune response during 2016 despite nutrient limitation. Although we did not specifically measure pathogen burden, exposure to parasites or other pathogens may increase during El Niño events (Marcogliese, 2008), and increased SST can broadly affect how pinnipeds may clear pathogens (Seguel *et al.*, 2018).

IgM was the only parameter to show an association with body length measured in juvenile CSL only. For pinnipeds, large body mass and adipose reserves are important for allocating increased energy stores towards heightened adaptive immune responses (Peck *et al.*, 2016). Here we find that shorter SL (a proxy for body size and age) is associated with heightened IgM. This is striking, as CSL pups in another study born during anomalous conditions in 2015 exhibited decreased body condition and both IgG and IgM responses (Banuet-Martínez *et al.*, 2017). Similarly, Galápagos sea lion pups with low body condition also had decreased IgG responses (Brock *et al.*, 2013b). This species lives in areas characterized by low productivity environments and pups may invest in immunity and growth according to scarce resources provided by their mother. The authors propose that, if resources are low, evident by low body condition, pups will likely allocate energy towards somatic growth over immune function. Because length and body condition do not always correlate, it is possible that larger SL in our study may actually be reflective of older age. Our data may suggest that older animals apportion some energetic reserves towards costly immune processes, such as IgM production, and fight off pathogens early during exposure rather than continue growth. Furthermore, it may be equally likely that older juveniles have prior exposure to many circulating pathogens, and thus would elevate IgM in response to novel pathogens. Available energy reserves in developing juvenile sea lions thus likely impact the allocation of energy towards immune response at the beginning of exposure to pathogens where the developmental costs of immune responses are high. Controlling for annual effects, we also found that females had higher IgM than males along with increased stress and thyroid hormones. As a sexually dimorphic species, it is likely that, even at an early stage, juvenile males are allocating greater resources towards somatic growth than females and may be disproportionally affected by energetic trade-offs to stress and immune responses.

### Cumulative ocean stressors

As large marine predators, sea lions respond dynamically to changes within their environment and provide clues about the current state of marine ecosystems (Simeone *et al.*, 2015). Our study highlights the importance of sea lions as sentinel species for changing marine ecosystems and provides reference ranges for several markers for acute stress, metabolism and immune function during anomalous conditions for both developing and mature sea lions. Here we expand stress data for this model species, which may importantly provide future comparisons and environmental context, and inform marine mammal conservation decisions (Atkinson *et al.*, 2015, Ocean Studies Board, 2017). The marine environment contains a multitude of stressors with which sea lions must physiologically cope. Our results may be equally confounded by documented naturally occurring toxic algal blooms, which also increase with SST (Van Dolah, 2000) and are known to cause domoic acid toxicosis and subsequent mortality in CSL (Gulland *et al.*, 2012). Chronic exposure to domoic acid will reduce endocrine function by potentially altering the HPA pathway, thereby reducing cortisol release (Gulland *et al.*, 2012). Natural stressors can be compounded by anthropogenic sources, such as exposure to marine debris, noise, contaminants and habitat modification or degradation (Kovacs *et al.*, 2012, Maxwell *et al.*, 2013), all of which can manifest as population-level disturbances to sea lions (McHuron *et al.*, 2018b). Thus, the ambiguity of multiple stressors within the marine ecosystem can make it difficult to disentangle the exact cause and the cumulative effects observed in our study animals. Although reactive stress responses to normal life history events or severe weather are likely adaptive (Boonstra, 2013), it is unclear how these responses may impact fitness and life history trade-offs (Zera and Harshman, 2001) for individual sea lions. Large-scale warming anomalies within the California Current ecosystem will likely be persistent (Peterson *et al.*, 2015); and as El Niño events are predicted to become more frequent (Cai *et al.*, 2014), individuals may not be able to effectively respond to increased frequencies of disturbances in novel or changing conditions (Wingfield, 2013). The United States population of CSL is currently deemed stable; however, recent models show that incremental increases in SST likely drastically reduce future populations (Laake *et al.*, 2018). Our results suggest that chronic stress due to oceanographic anomalies and nutritional limitations may alter physiological processes and has the potential to exacerbate natural stressors due to life history events.

## Supplementary Material

Supplementary_Figure_1_and_2,_R2_coz010

Supplementary_Figure_3_and_4,_R2_coz010

Supplementary_Tables_1_and_2,_ R2_coz010
