# Supplementary material for: Climate variability and life history impact stress, thyroid, and immune markers in California sea lions (Zalophus californianus) during El Niño conditions
Source: Conserv Physiol. 2019 May 15;7(1):coz010. doi: 10.1093/conphys/coz010 (PMC6518924; doi:10.1093/conphys/coz010)
Supplement: Supplementary_Figure_1_and_2,_R2_coz010 [file supplementary_figure_1_and_2,_r2_coz010.docx]

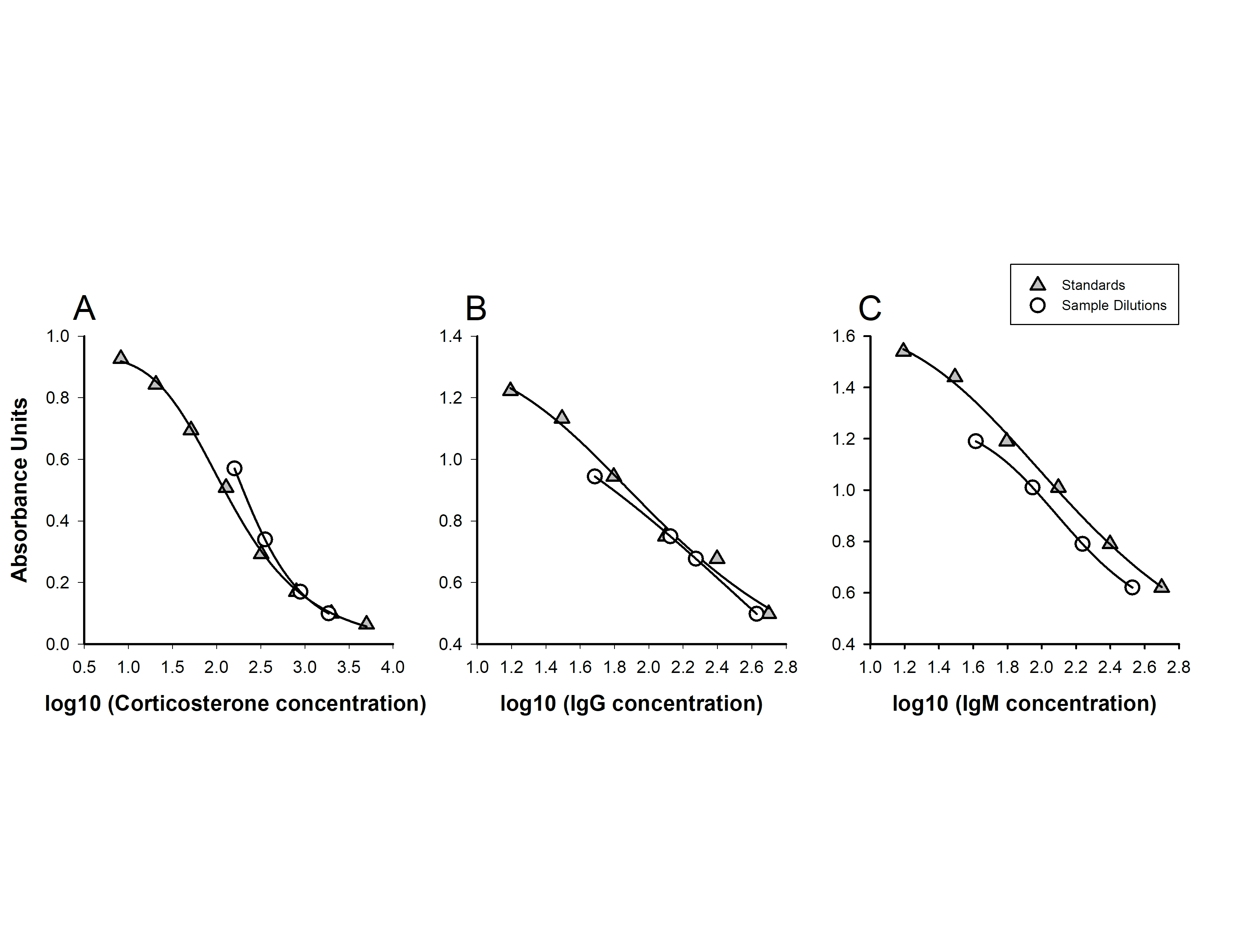
Supplementary Figure 1. Parallelism of mean absorbance values of serum sample dilutions with assay standard curves for corticosterone (A), IgG (B), and IgM (C) ELISA platforms.


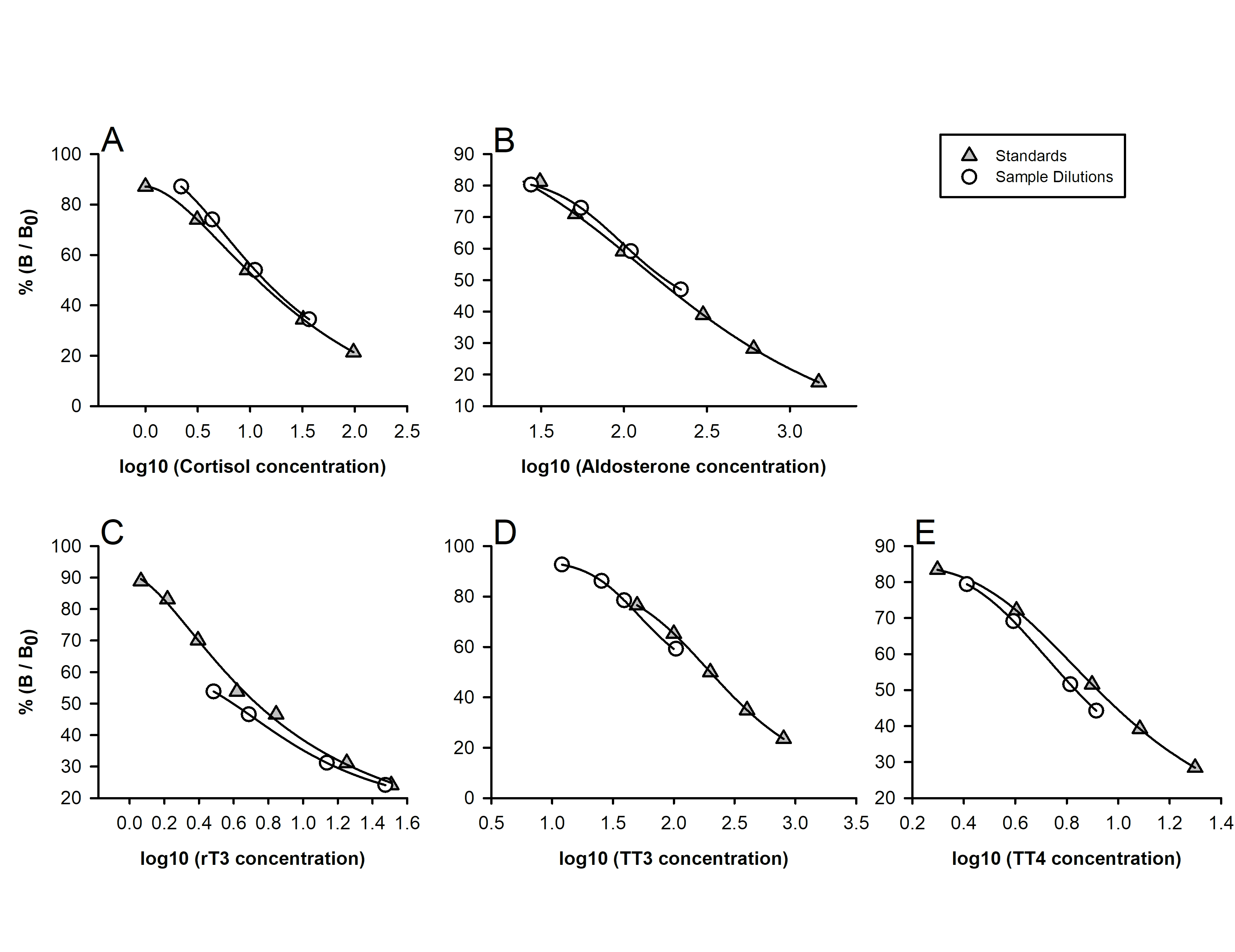


Supplementary Figure 2. Parallelism of % (B/B_0_) values of serum sample dilutions with standard curves for cortisol (A), aldosterone (B), rT3 (C), TT3 (D), and TT4 (E) RIA platforms.
