# Supplementary material for: Climate variability and life history impact stress, thyroid, and immune markers in California sea lions (Zalophus californianus) during El Niño conditions
Source: Conserv Physiol. 2019 May 15;7(1):coz010. doi: 10.1093/conphys/coz010 (PMC6518924; doi:10.1093/conphys/coz010)
Supplement: Supplementary_Figure_3_and_4,_R2_coz010 [file supplementary_figure_3_and_4,_r2_coz010.docx]

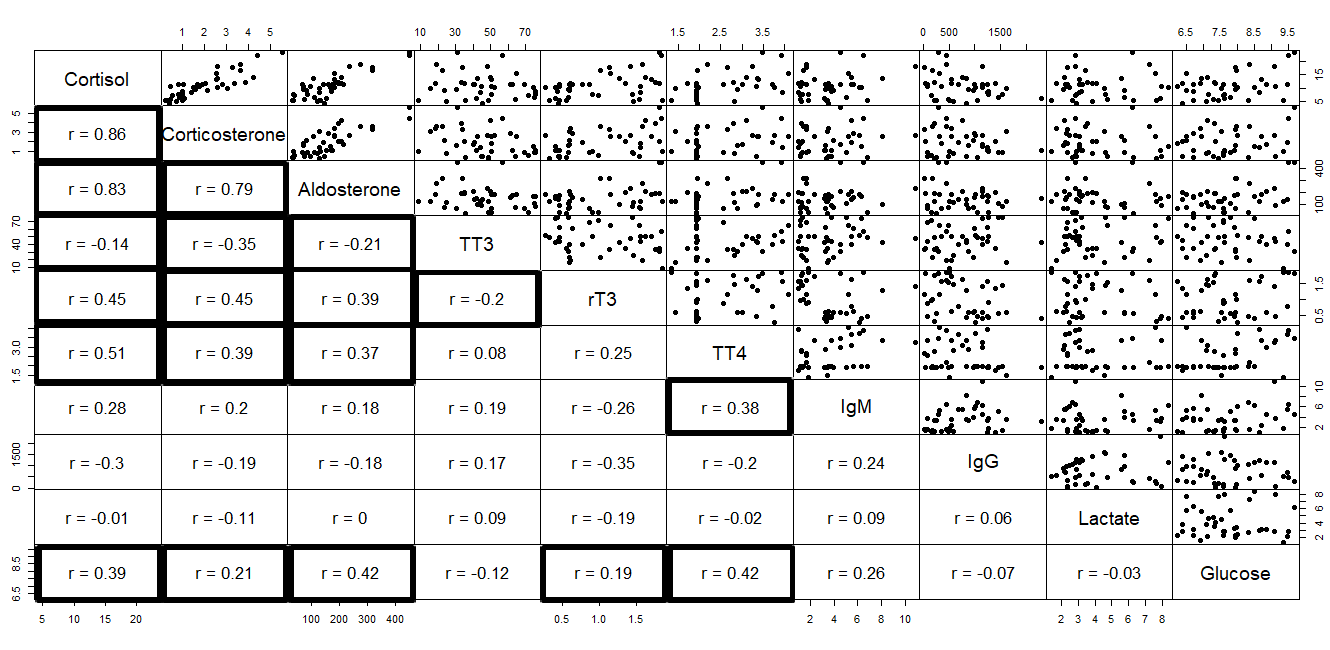


Supplementary Figure 3. Scatterplot matrix of all analytes measured in juvenile California sea lions using multivariate comparisons. Pearson’s correlation coefficients (r) for each significant relationship are outlined in bold in lower panels.


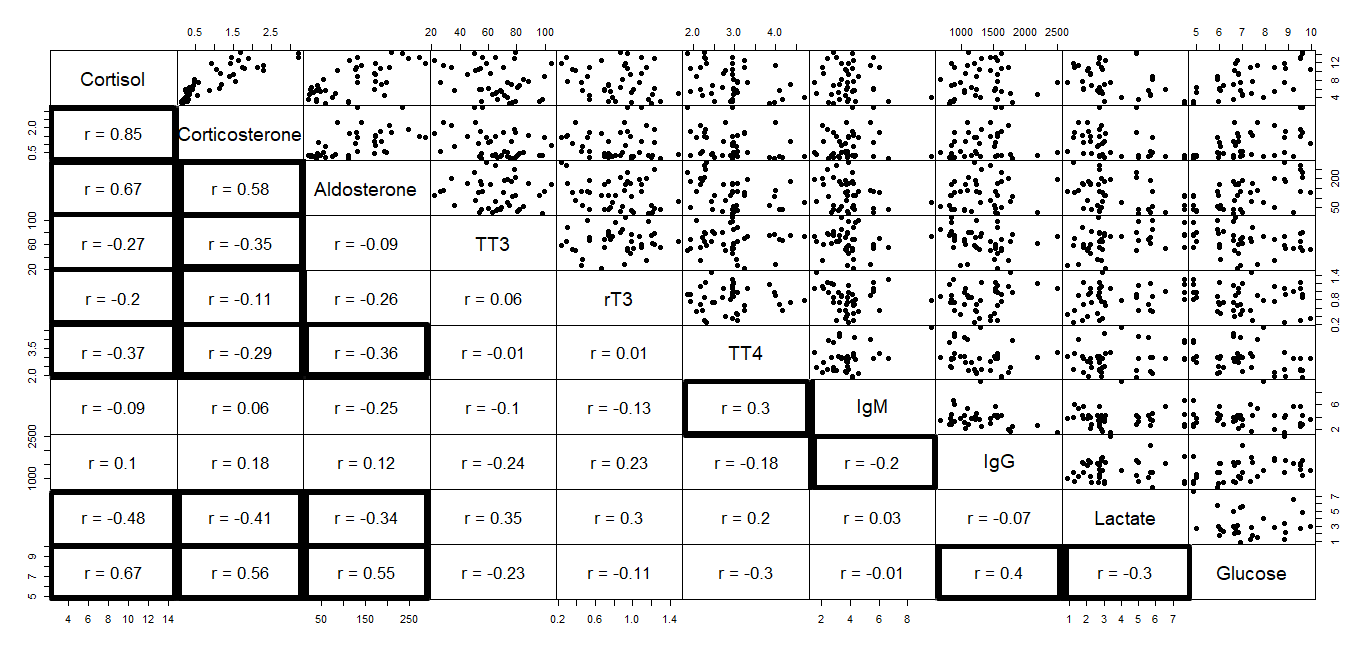


Supplementary Figure 4. Scatterplot matrix of all analytes measured in adult male California sea lions using multivariate comparisons. Pearson’s correlation coefficients (r) for each significant relationship are outlined in bold in lower panels.
