# Supplementary material for: Climate variability and life history impact stress, thyroid, and immune markers in California sea lions (Zalophus californianus) during El Niño conditions
Source: Conserv Physiol. 2019 May 15;7(1):coz010. doi: 10.1093/conphys/coz010 (PMC6518924; doi:10.1093/conphys/coz010)
Supplement: Supplementary_Tables_1_and_2,_ R2_coz010 [file supplementary_tables_1_and_2,_r2_coz010.docx]

Supplementary Table 1. Means ± standard deviations of concentrations of the analytes measured in 2015 and 2016 for juvenile (age 1-4) California sea lions grouped by sex= and year. Different letters denotes significant differences between groups, according to post-hoc student’s t-tests (*P <* 0.05).

|  | **Juvenile Female** | | **Juvenile Male** | |
| --- | --- | --- | --- | --- |
|  | **2015** | **2016** | **2015** | **2016** |
| Cortisol (µg/dL) | 14.7 ^a^ ± 4.6 | 9.14 ^c^ ± 2.5 | 12.1 ^b^ ± 4.3 | 8.05 ^c^ ± 2.76 |
| Corticosterone (µg/dL) | 2.70 ^a^ ± 1.36 | 1.23 ^b^ ± 0.78 | 2.48 ^a^ ± 1.3 | 1.14 ^b^ ± 0.83 |
| Aldosterone (pg/ml) | 239.3 ^a^ ± 114 | 114.2 ^b^ ± 50.0 | 186 ^a^ ± 97.6 | 106 ^b^ ± 58.6 |
| Total T4 (µg/dL) | 3.0 ^a^ ± 0.7 | 2.35 ^b^ ± 0.71 | 2.44 ^b^ ± 0.92 | 1.99 ^b^ ± 0.36 |
| Total T3 (ng/dL) | 38.8 ^a^ ± 17.0 | 65.9 ^b^ ± 13.0 | 24.1 ^c^ ± 16.1 | 41.1 ^a^ ± 15.4 |
| Reverse T3 (ng/dL) | 1.28 ^a,b^ ± 0.5 | 0.97 ^b,c^ ± 0.43 | 1.55 ^a^ ± 0.36 | 0.79 ^c^ ± 0.39 |
| IgG (mg/dL) | 503.9 ^a,b^ ± 389 | 1026 ^c^ ± 692 | 434.9 ^a^ ± 402 | 895.9 ^b,c^ ± 591 |
| IgM (mg/dL) | 5.41 ^a^ ± 3.7 | 2.61 ^b^ ± 1.9 | 2.07 ^b^ ± 1.16 | 2.91 ^b^ ± 1.66 |
| Glucose (mmol/L) | 8.46 ^a^ ± 0.9 | 7.73 ^b^ ± 0.7 | 7.95 ^b^ ± 0.9 | 7.35 ^c^ ± 0.71 |
| Lactate (mmol/L) | 3.41 ^a^ ± 1.8 | 3.65 ^a^ ± 1.7 | 4.23 ^a^ ± 2.3 | 4.18 ^a^ ± 1.8 |

Supplementary Table 2. Means ± standard deviations of concentrations of the analytes measured both pre- and post-breeding season in adult male California sea lions in 2016 grouped by time period. Different letters denotes significant differences between seasons, according to post-hoc student’s t-tests (*P <* 0.05).

|  | **Pre - Breeding** | **Post - Breeding** |
| --- | --- | --- |
| Cortisol (µg/dL) | 10.1^a^ ± 2.8 | 5.51 ^b^ ± 2.38 |
| Corticosterone (µg/dL) | 1.65 ^a^ ± 0.9 | 0.55 ^b^ ± 0.45 |
| Aldosterone (pg/ml) | 160.2 ^a^ ± 61.1 | 74.4 ^b^ ± 60.7 |
| Total T4 (µg/dL) | 2.79 ^a^ ± 0.5 | 3.05 ^a^ ± 0.66 |
| Total T3 (ng/dL) | 53.6 ^a^ ± 18.3 | 66.2 ^b^ ± 18.1 |
| Reverse T3 (ng/dL) | 0.80 ^a^ ± 0.35 | 0.89 ^a^ ± 0.23 |
| IgG (mg/dL) | 1459.5 ^a^ ± 410 | 1176.5 ^b^ ± 346 |
| IgM (mg/dL) | 3.27 ^a^ ± 1.46 | 3.67 ^a^ ± 1.78 |
| Glucose (mmol/L) | 8.12 ^a^ ± 1.21 | 6.45 ^b^ ± 1.14 |
| Lactate (mmol/L) | 2.38 ^a^ ± 1.0 | 4.11 ^b^ ± 1.8 |
